# Transferability of the robot assisted and laparoscopic suturing learning curves

**DOI:** 10.1007/s11701-023-01753-1

**Published:** 2024-01-27

**Authors:** E. Leijte, I. De Blaauw, C. Rosman, S. M. B. I. Botden

**Affiliations:** 1 Department of Surgery, Radboud University Medical Centre, Geert Grooteplein 10 Route 618, 6500HB Nijmegen, The Netherlands; 2 Department of Paediatric Surgery, Radboud University Medical Centre, Nijmegen, The Netherlands

**Keywords:** Simulation, Learning curve, Minimally invasive

## Abstract

Robot assisted surgery (RAS) is increasingly used, and besides conventional minimally invasive surgery (cMIS) surgeons are challenged to learn an increased array of skills. This study aimed to assess the influence of both learning curves on each other. A prospective randomized crossover study was performed. Participants without cMIS or RAS experience (Groups 1 and 2), and cMIS experienced, (Group 3) were recruited. Three suturing tasks (intracorporal suturing, tilted plane and anastomosis needle transfer) were performed on the EoSim cMIS simulator or RobotiX RAS simulator up to twenty repetitions. Subsequently, Groups 1 and 2 performed the tasks on the other modality. Outcomes were simulator parameters, validated composite and pass/fail scores. In total forty-three participants were recruited. Overall RAS suturing was better in Group 1 (cMIS followed by RAS tasks) and 3 (RAS tasks) versus Group 2 (RAS followed by cMIS tasks) for time (163 s and 157 s versus 193 s *p* = 0.004, *p* = 0.001) and composite scores (92/100 and 91/100 versus 89/100 *p* = 0.008, *p* = 0.020). The cMIS suturing was better for Group 2 versus 1 (time 287 s versus 349 s *p* = 0.005, composite score 96/100 versus 94/100 *p* = 0.002). Significant differences from the RAS suturing pass/fail were reached earlier by Group 3, followed by Groups 1 and 2 (repetition six, nine and twelve). In cMIS suturing Group 2 reached significant differences from the pass/fail earlier than Group 1 (repetition four versus six). Transferability of skills was shown for cMIS and RAS, indicating that suturing experience on cMIS or RAS is beneficial in learning either approach.

## Introduction

After the introduction of robot-assisted surgery (RAS) with the DaVinci system (Intuitive Surgical Inc. USA), the frequency and application of the usage of RAS has been growing continuously [1]. With the field of general surgery as the most recent growing surgical specialism, residents in training and specialized surgeons, which are often highly skilled in conventional minimally invasive surgery (cMIS), are being challenged to attain and maintain technical skills in both methods [2]. This is especially the case for residents in training who are novices and need to acquire proficiency in both techniques. Additionally, despite the increasing implementation of RAS in clinical practice, operative participation of residents is limited, because a dual console is lacking frequently [3]. This leads to an increasing demand for validated and standardized RAS curricula and expresses the importance of simulation training to acquire competency outside the clinical setting [4–7]. Skills acquisition of RAS simulation training has been shown to be faster compared to cMIS simulation training for novices [8–10]. There is still discussion on the level of skill transfer between both modalities, although there are similarities between RAS and cMIS. There are several studies performed which have found a transfer effect from cMIS to RAS [2, 11–17]. However, the transferability effect of RAS on cMIS is more unclear with three studies showing no effect and two reporting a positive transfer effect [11, 13, 15–17]. Most of these studies concerned basic peg transfer skills [12–16] or studied only the initial part of a learning curve with limited number of repetitions [2, 11, 12, 14, 17], whereas the main benefit of RAS is expected during the performance of complex skills such as suturing. Therefore, this study aims to assess the skills transferability of cMIS and RAS during a suturing learning curve in a crossover simulated setting. With this study we aim to give further insight in complex skill acquisition to improve competence-based training for surgical residents.

## Methods

### Participants

Participants were recruited voluntary at Radboud university medical centre Nijmegen, The Netherlands. To include participants with a basic level of surgical understanding of the concept of cMIS and RAS but without experience in these techniques, medical interns and physician researchers were recruited for either Group 1 or 2. After recruitment, allocation was based randomly on the basis of the current simulator availability without stratification. To include participants experienced with cMIS but inexperienced with RAS, surgical residents and surgeons were included in Group 3. Due to the non-medical setup and voluntary participation of this study, no ethical board approval was required.

### Simulators

#### EoSim cMIS AR simulator

**Fig. 1:**
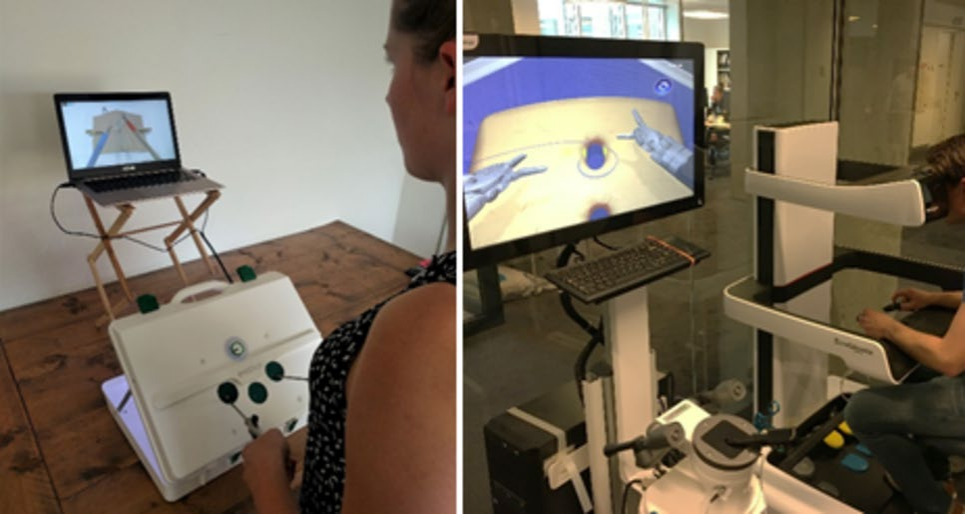
The EoSim laparoscopic augmented reality simulator (left) and the RobotiX robot-assisted virtual reality simulator (right) as used in this study

To compare instrument parametric outcomes of both modalities the EoSim minimally invasive surgical augmented reality (AR) simulator (Eosurgical ltd., Edinburgh, Scotland, UK) was used in this study (Fig. 1). Previous validation studies reported evidence of multiple aspects of validity for this simulator [18–22]. The EoSim setup consisted of a suitcase model the participants performance displayed on a connected laptop with the provided Surgtrac software. To assure correct positioning of the screen an adjustable standard was used. The simulator parametric outcomes consisted of the parameters; total time, distance, working space and off-screen (Table 1). Additionally, a researcher noted the number of sutures made during the third task to determine the time per suture and instrument distance per suture.

**Table 1:** Parameter definitions and included parameters of the used composite scores

General parameters		Definitions	
Total time		Task time in seconds	
Instrument distance		Total instrument distance in meters	
EoSim parameters			
Off-screen		Sum of the off-screen percentage for each instrument (left and right)	
Working space		Average distance between the instruments in centimeter	
RobotiX parameters			
Needle drops		Number of times the needle was dropped	
Unnecessary piercing		Number of times the needle was inserted and retracted through the same entry point	
Total errors		Sum of errors occurred during the task such as instrument collisions and unnecessary piercing	
Off-screen		Number of times the instruments were out of view	
Instruments collisions		Number of times the instruments collided	
Composite scores			
cMIS	Task 1	Time and distance	
	Task 2	Time, distance, working space and off-screen	
	Task 3	Time per suture, distance per suture	
RAS	Task 1	Time, suture breakage, needle drops and unnecessary piercing	
	Task 2	Time, total distance, total number of movements, inaccurate punctures, instrument collisions and needle precision	
	Task 3	Time, distance left instrument, instruments collisions and precise needle passages	

#### RobotiX RAS VR simulator

For the robot-assisted tasks the RobotiX virtual reality (VR) simulator was used (Fig. 1) which showed evidence for validity in previous studies on multiple aspects [23–30]. The system, consists of a main console for a trainee and a control tower, 3D Systems (3D Systems Inc., Cleveland OH). Participants were able to adjust working height and eye level of the main console. The ‘Mentorlearn’ software kept track of each individual performance and progress of the custom-constructed learning curve curriculum used for this study. The software parametric outcomes consisted of multiple parameters which were divided into movement, safety and task-specific parameters as shown in Table 1.

### Tasks

To simulate the required skills during complex suturing the tasks for this study were selected to represent the separate components. Therefore, Task 1 consisted of an intracorporal suture and knot tying task, Task 2 consisted of a tilted plane needle transfer and Task 3 of an anastomosis needle transfer task. The cMIS Tasks were previously developed and validated [20]. Further specification of the RAS and cMIS tasks are described in the supplementals of a previously performed learning curve study [8]. The third task was selected to support the skill acquisition of Tasks 1 and 2. Therefore, only a limited amount of repetitions (each repetition consisting of eight to ten needle transfers) were performed.

### Protocol

**Fig. 2:**
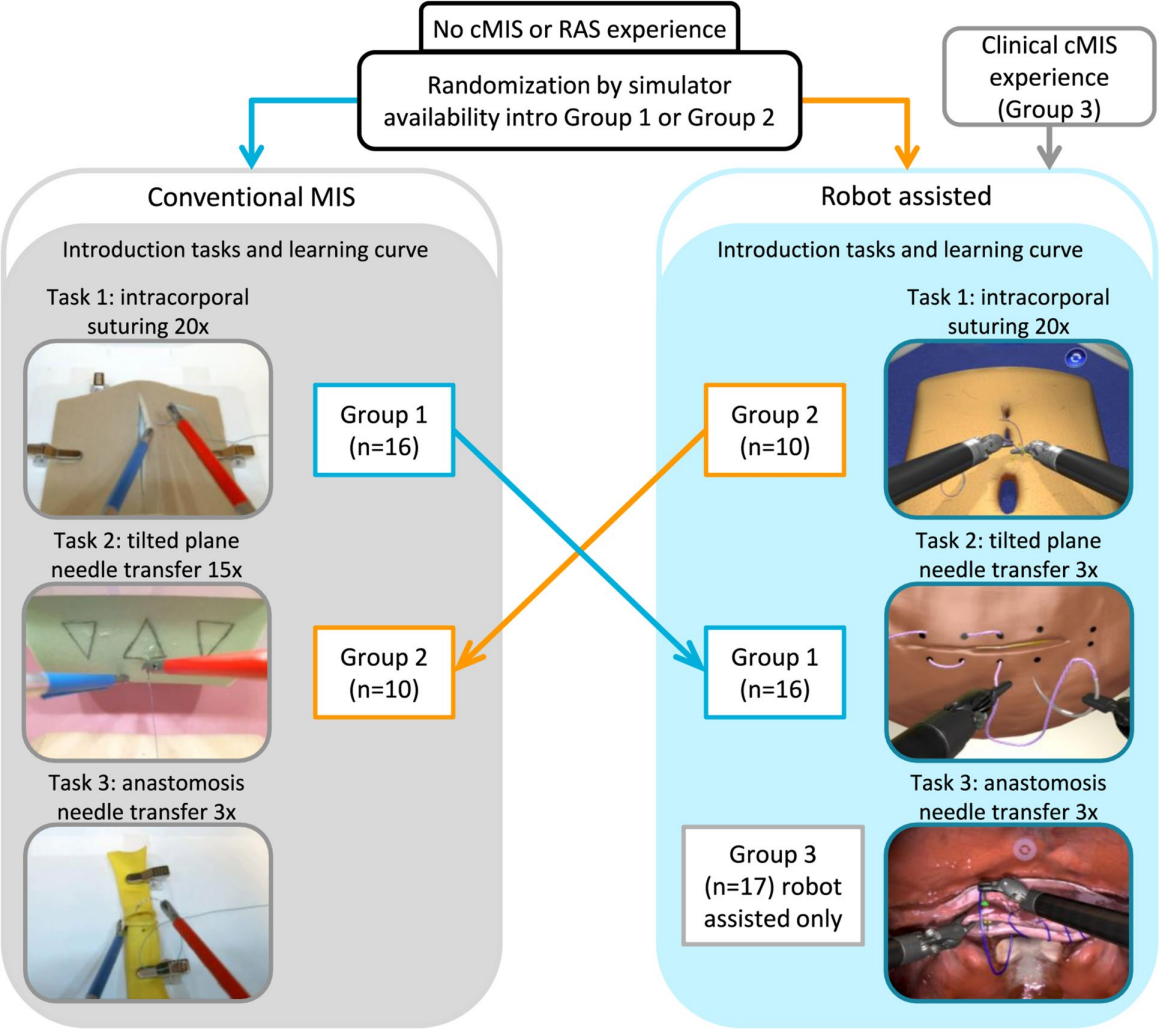
Flowchart of the study protocol and of the performed tasks on the EoSim and RobotiX

After recruitment, all participants completed a questionnaire regarding their informed consent, demographic information and surgical experience. Depending on the available simulator participants received an instruction and subsequently performed two familiarization tasks on either the EoSim or the RobotiX as shown in Fig. 2. Participants in Group 1 started with tasks on the EoSim followed by the RobotiX simulator and participants in Group 2 started with the RobotiX followed by the EoSim simulator. Participants in Group 3 only performed tasks on the RobotiX simulator. This group was included to assess the difference between clinical cMIS experience and simulator cMIS experience. After accomplishing familiarization tasks, the repetitive training of the suturing tasks was performed under supervision of a researcher up to a maximum of one hour and divided in multiple sessions. The researcher kept track of each participants progress and data storage. The intracorporal suturing (Task 1) training was regarded complete after twenty repetitions or three consecutive similar performances in a row based on the time parameter. The number of repetitions was based on a previous study by Botden et al. showing a suturing learning curve in fifteen repetitions on a laparoscopic simulator [31]. Therefore, twenty repetitions were used to achieve a substantial part of the learning curve. Due to the high visual and parametric similarity on both systems Task 1 was used as the ‘main task’ for learning curve evaluation. Task 2 was completed for the cMIS learning curve after fifteen repetitions and for RAS after three repetitions (one repetition consists of five suture transfers). Task 3 was regarded complete after three repetitions on both the EoSim and RobotiX simulator. After completion of the required repetitions on either cMIS or RAS the participant switched to the other modality, where they performed the suturing tasks after the instruction and introduction tasks. The main outcome of this study were the calculated composite score for each task. Secondary outcomes were the time, safety and movement parameters.

### Statistical analysis

The statistical analysis was performed using Statistical Package for Social Sciences (SPSS) software version 25 (IBM Corp., Armonk NY). All p-values of < 0.05 were considered statistically significant. Based on a previous RAS validation study the power analysis resulted in a minimal of eight participants per group to demonstrate a difference RAS composite score of fourteen points (Means 87 versus 73, Standard deviation 10, β = 0.2 and α = 0.05) [32]. Composite scores for the cMIS tasks were calculated using data from a previous study where construct validity was shown for several parameters per task (Table 1) [20]. The cMIS composite scores were calculated after normalization of the parameters showing construct and range from 0 to 100 with 100 being the best score. Accordingly, a cut-off score was calculated using the contrasting groups analysis model from the study by Jørgenson et al. [33]. This analysis calculates the optimal cut-off value (lowest false positive and false negative ratio) between novice and expert users. For the RAS tasks the composite scores were based on the validated parameters shown in Table 1 [32]. Additionally, the suturing composite scores were compared to the calculated cut-off value from the contrasting groups analysis using a one-sample t-test. Differences between two groups were calculated using the independent t-test and a one-way ANOVA was used when comparing three groups.

## Results

### Demographics

A total of 43 participants completed this study as shown in Table 2. Group 1 (*n*** = **16) and Group 2 (*n*** = **10) consisted of medical students and physician researchers with a mean age of 23 and 24 years, respectively. The laparoscopic experienced group (Group 3, *n*** = **17) consisted of thirteen residents in training and four surgeons. The specialties in group 3 ranged from abdominal/oncological surgery to urology, gynaecology and paediatric surgery participants. The difference in group size was due to the difference in availability of the simulators as participants in Group 3 only performed the RAS simulation tasks. The total minutes Group 1 and Group 2 spent of the performance of all three task showed a minor difference of 19 min (*p* = 0.514).

**Table 2:** demographics of the included participants

			Group 1 (* n*** = **16)	Group 2 (* n*** = **10)	Group 3 (* n*** = **17)	Total (* n*** = **43)
Mean age (SD)			23 (1.6)	24 (2.0)	34 (4.6)	28 (6.4)
Sex (male/female)			6/10	5/5	10/7	21/22
Dexterity (right/left)			15/1	7/3	16/1	38/5
Total task training minutes (SD)			327 (51)	308 (78)	83 (19)	
Surgical skill level	Medical student		16	10	0	26
	Resident in training	1–2 years	–	–	2	2
		3–4 years	–	–	9	9
		5–6 years	–	–	2	2
		Surgeon	–	–	4	4
Specialty		None	16	10	0	26
		Surgery	–	–	4	4
		Pediatric	–	–	3	3
		Urology	–	–	2	2
		Gynecology	–	–	8	8
Laparoscopic experience	Overall	0 years	16	10	0	26
		> 0—5 years	–	–	14	14
		6—10 years	–	–	1	1
		> 10 years	–	–	2	2
	Basic procedures	0	16	10	0	26
		> 0—30	–	–	5	5
		31—100	–	–	7	7
		> 100	–	–	5	5
	Advanced procedures	0	16	10	4	30
		> 0—10	–	–	7	7
		11—50	–	–	5	5
		> 50	–	–	1	1

SD: standard deviation. Procedures such as appendectomy or cholecystectomy were considered basic. Procedures with intracorporal suturing such as fundoplication were considered advanced

### Learning curve progression

The learning curve progression was based on the intracorporeal suturing task (Task 1). During the cMIS learning curve, participants performed at least sixteen suture repetitions in Group 1 and eighteen in Group 2. The complete twenty repetitions were performed by nine participants in Group 1 and seven in Group 2. The twenty suturing repetitions during the RAS learning curve was completed by twelve participants in Group 1, eight in Group 2 and ten in Group 3. The participants completed at least eighteen repetitions in Group 1 and nineteen repetitions in Group 2 and Group 3. During cMIS Task 2 at least ten repetitions were completed by Group 1 and twelve by Group 2. The complete fifteen repetitions were accomplished by twelve participants in Group 1 and seven in Group 2. The three repetitions required to complete RAS Task 2 and 3 learning curves were achieved by all participants in Group 2 and Group 3. Due to limited time for training one participant in Group 1 was unable to perform the third repetition of RAS Task 2 and 3 but was included in the analysis. The cMIS Task 3 was completed by all participants.

### Effects cMIS training on RAS performance

#### Overall

**Fig. 3:**
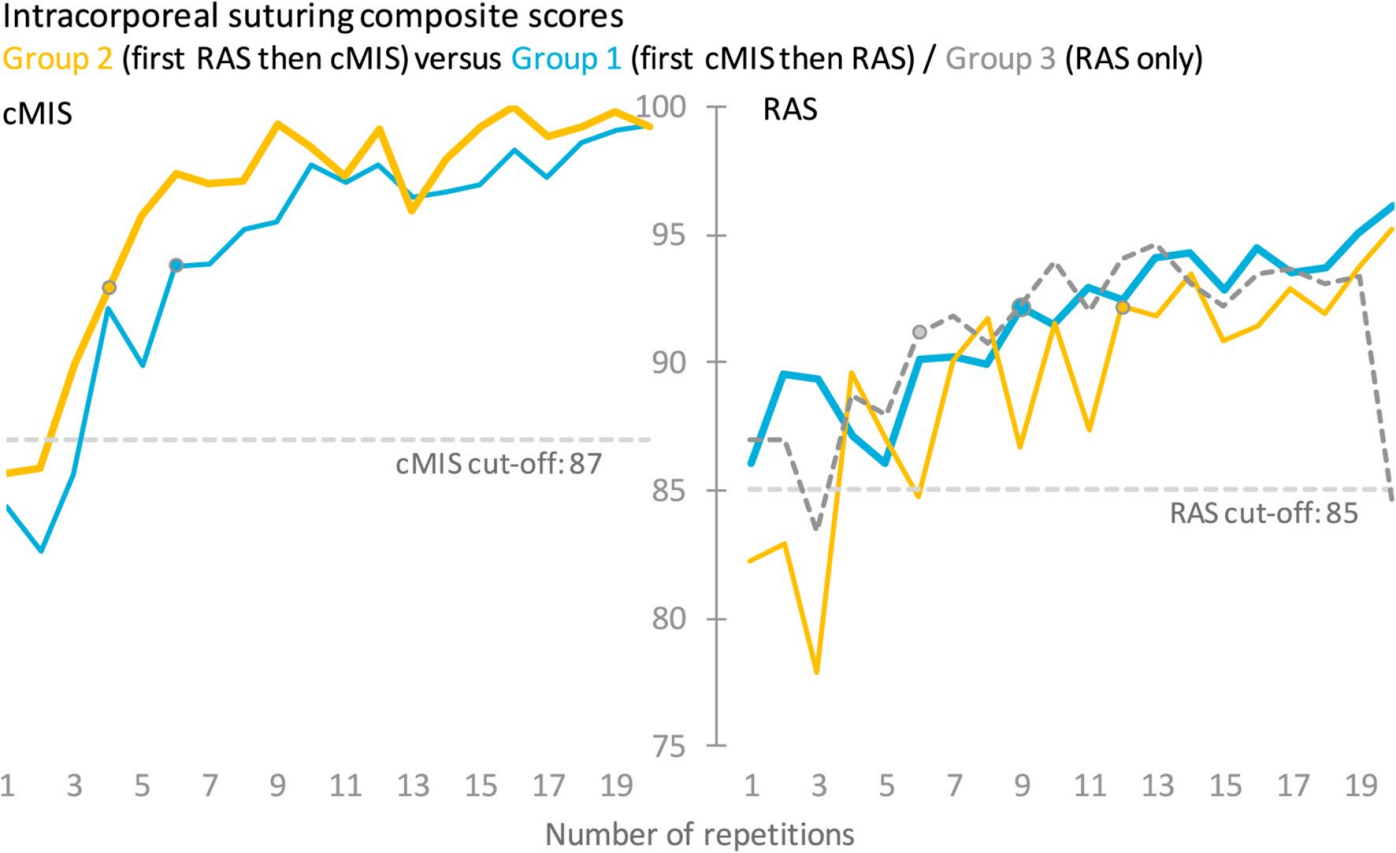
Mean composite score results of the intracorporal suturing task of the cMIS and RAS learning curves. Overview of composite scores is shown in Appendix 1

The RAS intracorporal suturing task resulted in a beneficial effect in a composite score and task time from the cMIS simulator (Group 1) or previous clinical laparoscopic experience (Group 3) as shown in Table 3 and Fig. 3. Comparing Group 2 to Groups 1 and 3, a significantly longer task time (*p* = 0.004 and *p* = 0.001) and a lower composite score was found (*p* = 0.008 and v0.020). The participants in Group 3 only showed a statistically significant faster performance time for Task 3 versus Group 1 (314 s versus 393 s, *p* = 0.021). No significant beneficial effect in task time or composite score was shown between participants in Group 2 versus Groups 1 or 3.

**Table 3:** Overall mean (SD) time and composite score outcomes per task and group

cMIS	Intracorporal suturing		Tilted plane		Anastomosis	
	Composite score	Time (s)	Composite score	Time (s)	Composite score	Time (s)
Group 1 (first cMIS then RAS)	94.2	349	73.7	270	73.7	1141
	(7.9)	(258)	(7.1)	(305)	(15.0)	466
Group 2 (RAS first then cMIS)	96.2	287	72.8	258	81.3	965
	(6.4)	(206)	(7.8)	(339)	(20.0)	(612)
p-value						
Group 1 vs 2	*0.002*	*0.005*	0.215	0.713	0.060	0.154
RAS						
Group 1 (first cMIS then RAS)	91.5	163	60.8	345	71.5	393
	(7.9)	(98)	(14.6)	(124)	(14.0)	(205)
Group 2 (RAS first then cMIS)	89.2	193	58.3	345	74.5	384
	(9.8)	(123)	(18.7)	(126)	(9.9)	(198)
Group 3 (RAS only)	91.0	157	62	309	74.6	314
	(10.2)	(108)	(14)	(105)	(9.0)	(105)
p-value						
Group 1 vs 2	*0.004*	*0.004*	0.516	0.996	0.309	0.846
Group 1 vs 3	0.531	0.419	0.633	0.118	0.194	*0.021*
Group 2 vs 3	*0.040*	*0.001*	0.330	0.169	0.950	0.084

Data in this table represents an overall mean (standard deviation) of task time and composite score per task. P-values were calculated using an independent t-test with a p-value of < 0.05 being considered statistically significant

#### RAS intracorporeal suturing

**Fig. 4:**
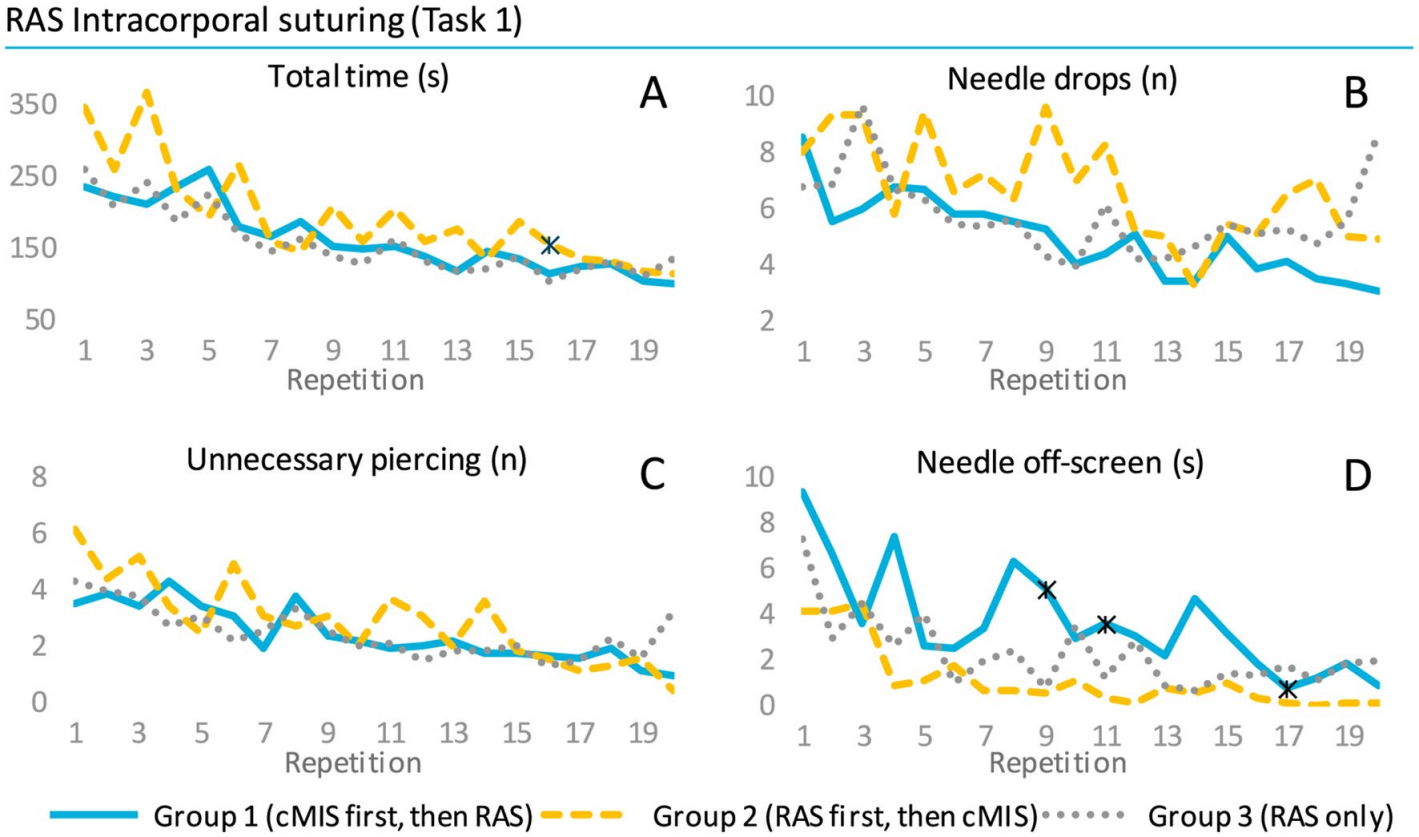
Mean performance outcomes of the intracorporal suturing task of the RAS* p* =  learning curve. * indicates a p-value < 0.05 between Group 1 and 2

**Fig. 5:**
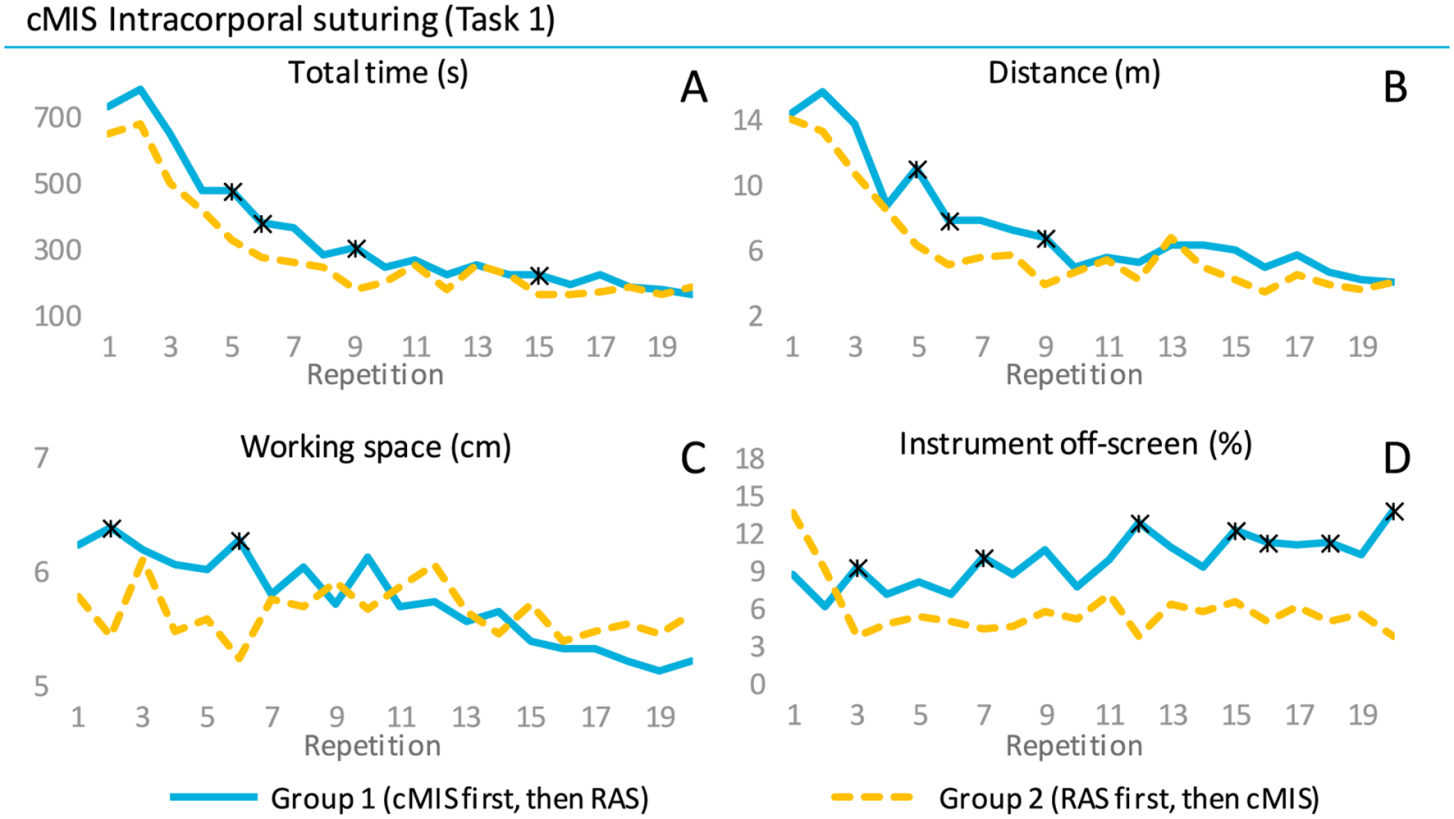
Mean performance outcomes of the intracorporal suturing task of the cMIS learning curve. * indicates a p-value < 0.05 between Group 1 and 2

The RAS suturing task appeared to be completed in less time and with a higher composite score by participants in Group 1 compared to Group 2 (Fig. 4 and 5). However, statistically significant differences between Groups 1 and 2 were only evident for task time at repetition sixteen (113 s versus 155 s, *p* = 0.030) and for the composite score at repetition three (89 versus 78, p = 0.017, see Appendix 1). Group 3 showed similar performance as Group 1 and was statistically different in task time at repetition sixteen (93 versus 91, p = 0.013). A significant difference from the cut-off value was reached first by Group 3 at repetition six, then by Group 1 at repetition nine and finally by Group 2 at repetition twelve. The needle handling was on average scored better by Group 1 with less needle drops compared to Group 2, however, without any statistically significant difference. The needle was held significantly more off-screen by Group 1 versus Group 2 during repetition nine (5.1 s versus 0.6 s, p = 0.016), eleven (3.6 s versus 0.4 s, p = 0.047) and seventeen (0.8 s versus 0.04 s, p = 0.026).

#### RAS tilted plane and anastomosis needle transfer

Overall results of Task 2 and 3 are shown in Table 3. The tilted plane needle transfer (Task 2) was completed by all groups with similar results on a composite score and time resulting in no significant differences. The anastomosis needle transfer (Task 3) was performed best by the participants with cMIS experience (Group 3) with the highest composite score and the lowest time results. However, only the time parameter showed a statistically significantly difference compared to Group 1 (*p* = 0.021). with an overall time difference of 79s.

### Effects RAS training on cMIS performance

#### Overall

Performance results from the overall scores are shown in Table 3. During the cMIS intracorporal suturing task (Task 1) a possible beneficial effect of RAS simulator experience is shown for Group 2 with an overall statistically significant higher composite score of 2 points (*p* = 0.002) and 62 s faster task time (*p* = 0.005). Similar results for the overall outcomes are shown for Task 2 and 3. Group 2 shows a mean faster performance for Tasks 2 and 3 and a higher mean composite score at Task 3, although not statistically significant.

#### cMIS intracorporeal suturing

The suturing task was performed by Group 2 with a higher composite score and less time during the first ten repetitions (Fig. 3 and 5). Statistically this was shown to be significantly different in composite score compared to Group 1 for repetitions five (79 versus 88, *p* = 0.023), six (85 versus 91, *p* = 0.024), seven (85 versus 90, *p* = 0.046) and nine (88 versus 94, *p* = 0.025), see Appendix 1. In terms of task time Group 2 also showed statistically significantly better performance for the repetitions five (477 s versus 323 s, *p* = 0.036), six (380 s versus 278 s, *p* = 0.023), nine (309 s versus 180 s, *p* = 0.010) and fifteen (226 s versus 167 s, *p* = 0.034). Additionally, Group 2 showed an earlier significant difference from the cut-off value by two repetitions (Repetition 4 versus 6). The distance and working space parameters were performed with a steeper curve by Group 2 in the first half of the learning curve with multiple statistically significantly different repetitions. The participants in Group 2 reached early a stable plateau curve on the off-screen parameter compared to Group 1 with seven repetitions showing a statistically significant difference from which five range between repetition 12 and 20.

#### cMIS tilted plane and anastomosis needle transfer

Task 2 was performed equally overall by both groups shown in Table 3. The anastomosis needle transfer (Task 3) however, was performed with higher outcomes by Group 2 with a higher composite score and shorter time results. Although, no statistically significant difference could be found between groups.

## Discussion

This study shows a transferability effect from cMIS to RAS and vice versa. During cMIS training the RAS experienced participants showed a faster composite score differentiation from the pass/fail value, an initial steeper decrease in time and a better off-screen performance. This indicates that RAS experience leads to faster competency and safer performance during cMIS suturing tasks. The participants with cMIS training showed an earlier significant difference from the pass/fail value and were significantly better in the overall time and composite score results compared to RAS novices. However, cMIS experience showed a negative effect on the off-screen performance during RAS training, which emphasizes this as a critical parameter to be monitored during RAS training. The clinical cMIS experience was shown to distinguish earlier from the RAS pass/fail score compared to cMIS training experience but did not show other major differences in the performance outcomes.

The results of this study support previous findings regarding the transferability of skills from cMIS to RAS [2, 11, 12, 14–17], although these mainly focussed on basic skills. The study by Thomaier et al. showed a beneficial effect of cMIS or RAS training of a peg transfer task on both cMIS and RAS performance by novices [15]. Interestingly, the results of Thomaier et al. also found that after cMIS training there was no improvement in the off-screen parameter between RAS baseline and post-training performance. Whereas, the RAS training group showed a better improvement in the instruments off-screen parameter. These results indicate the importance of the off-screen parameter to be monitored for RAS trainees with cMIS experience as the lack of haptic feedback and off-screen instruments could cause unwanted tissue damage.

The skill transfer of RAS to cMIS was previously not found [11, 13, 16]. Whereas, the studies by Thomaier and Obek et al. did report a positive transfer effect from RAS to cMIS [15, 17]. These discrepancies are likely to be explained by the limited amount of repetitions or tasks used. This study showed the most positive skill transfer of RAS to cMIS, indicating that the more “intuitive” setting of RAS could be used for novice trainees to improve the initial difficulty of the cMIS learning curve. Our study is the first to examine a learning curve of multiple suturing tasks for up to twenty repetitions. Additionally, this study is strengthened by the randomized crossover setup and includes a separate group of participants with clinical cMIS experience. As stated by Kassite et al. the assessment of a learning curve should be based on multiple parameters [34]. In agreement with this statement, this study included multiple parameters, used in the composite scores and separately.

However, this study also has some limitations. At our institution, there was no availability of a DaVinci system for training purposes. This limited the possibility of matching the suturing tasks in detail on both modalities. However, the main goal of this study was not to compare the cMIS versus the RAS skill improvement but to assess the transferability of skills for which this setup worked well. Another limitation is the difference in group size Group 1 (*n*** = **16) and Group 2 (*n*** = **10) which was based on the simulator availability during training times. However, Group 3 (*n*** = **17) was only trained on the RAS simulator and, therefore, more participants were included according to protocol in the cMIS first group.

## Conclusion

The results of this study showed a transferability effect of skills from cMIS to RAS and from RAS to cMIS. The experience in either cMIS or RAS resulted in a faster differentiation from the used pass/fail score on the contrasting modality. Additionally, the RAS experience had a positive effect on the off-screen performance and could be beneficial for the learning of safer cMIS performances. These results indicate that previous cMIS or RAS experience shortens the learning curve and a mixed approach of RAS and cMIS training could be used when learning either of the modalities.

## Data Availability

The data that support the findings of this study are available from the corresponding author upon request. Data are located in controlled access data storage at the Radboud University Medical Centre.
